# Recherches Anatomico-Pathologiques sur l'Encephale, &c. or Anatomico-Pathological Researches on the Brain and Its Appendages

**Published:** 1824-09-01

**Authors:** 


### MEDICO-CHIRURGICAL

### REVIEW.

Vol. V.] Analytical Series. [No. 18.

" Nec tibi quid liceat sed quid fecisse decebit
" Occurrat, mentemque domat respectus honesti."

Vol. I.] SEPTEMBER 1, 1824. [No. 2.

[NEW SERIES.]

### I.

### LALLEMAND ON THE BRAIN.

*Recherches Anatomico-pathologiques sur l'Encéphale et ses Dependances.* Par F. Lallemand, Professeur de Clinique, &c. a Montpellier. Lettre Quatrìeme. 8vo. pp. 230. Paris, 1823.

" Ars Medica tota in Observationibus."—Hoffman.

OUR readers are aware that we have given full analyses of the three preceding letters of the distinguished pathologist of Montpellier;* and we now proceed to render an account of the present letter, which is principally occupied with an interesting class of affections, hitherto but little treated of in this country —namely, diseases of the brain and its coverings, propagated from inflammatory affections of external parts, especially the ear. We suspect that these affections are of much more frequent occurrence than is generally imagined, and that many lives are lost, from considering them as unimportant and external at the beginning, while, in reality, they are spreading to and invading structures of vital consequence within the head. On this account it must be instructive to have as many histories registered as possible, both to peruse, at present, and refer to hereafter, when similar accidents occur in practice.

* Vide No. 11. for December 1822.

In the present letter, then, our author proposes to treat of what may be termed *encysted abscesses*, which he conceives to be the result of acute, but much more frequently of chronic, inflammation. In the *former* case the train of symptoms is much more regular, and their character more marked than in the *latter*. In the *former*, the intensity of the malady overcomes age, sex, and temperament, which, in the chronic affections, modify and greatly obscure both the march and nature of the symptoms. It is only, as our author properly observes, by a careful collection and comparison of individual cases, that a proper idea can be formed of this class of complaints, and, therefore, the reader must divest himself of fastidiousness while perusing the narratives we are about to lay before him, and which we shall endeavour to condense into as small a space as possible.

*Case* 1. This case was communicated to our author by that very accurate pathologist and able surgeon M. Breschet. Mary Gabriel, from her youth, had something remarkable in her physiognomy. Whenever she took strong exercise, her face became red, and her respiration (which was habitually short) much embarrassed. At the age of forty-seven she ceased to menstruate, and began to complain of palpitations, and acute pain in the precordial region. She would frequently stop to feel the action of the heart, and prognosticated that she would soon die. Her face now became of such a blue cast, even when walking slowly, that she was ashamed of appearing in the streets. She was subject to profuse nasal hæmorrhages—cramps in her limbs—debility. On the 1st July 1821, she complained to her sister of cramp and pain in the *left* hand and foot, and soon experienced much embarrassment in the motion of these members, which, in a short time, was entirely lost, as were sensation and motion of the whole body. On the 3d, she entered the hospital, her face being of a violet colour, eyes sparkling and prominent, respiration very difficult, pulse small and easily compressible at the *left* wrist, but hard and strong in the right arm; complete loss of sensation and motion of the *left* side of the body. In the night, the paralysed members were suddenly affected with convulsive movements, and the breathing became very embarrassed—the pulsations of the heart were very tumultuous. Fifteen leeches to the anus—digitalis internally. In the morning, the symptoms were calmer. 4th. Bled from the arm. Between the 5th and the 12th, the patient presented several paroxysms similar to those described on the 1st day; but, towards 12 o'clock on the 12th, she became insensible to surrounding objects, and agitated by convulsive movements, in which state she continued till the next day, when she expired.

*Dissection.* In the anterior part of the *right* hemisphere of the brain, an abscess was discovered, containing about three ounces of greenish pus, enclosed in a real cyst, capable of being completely dissected out, and yet the most accurate examination by glasses could not detect any vessel going between the brain and the cyst. The cerebral substance in contact with the cyst was of a deep red colour, which insensibly diminished as the distance from the cyst increased.

Throughout the whole of this coloured portion, the cerebral substance was less consistent than in the rest of the brain. The whole capillary system of the brain, arterial and nervous, was gorged with blood, and between the meninges was scattered some gelatinous matter here and there. The heart was greatly enlarged. The right auricle was very much so, and contained several ounces of blood. A small communication existed between the two auricles in the situation of the original foramen ovale. The auriculo-ventricular opening between the right auricle and ventricle was very much narrowed, the ventricle of this side not being more than the size of a pigeon's egg, nor capable of containing more than a few drachms of blood. The parietes of this ventricle were from 11 to 16 lines in thickness. At the origin of the pulmonary artery, instead of the three semilunar valves, there was one horizontal valve stretched across, with a hole in its centre. The left auricle was natural, the left ventricle rather enlarged, and some patches of ossification on the aorta.

M. Lallemand makes several pathological and physiological observations on the above case. He conceives that the malformation at the origin of the pulmonary artery was probably congenital, and by obstructing the flow of blood from the ventricle, caused, at each ventricular contraction, a reflux of blood into the right auricle, and thus a stasis in the whole venous system. On the other hand, some black blood was necessarily mixed with the red, through the foramen ovale, thus producing the blue tint of skin before described, together with the debility and the embarrassment in breathing when taking exercise.

The obstacle at the origin of the pulmonary artery explains the hypertrophy of the right ventricle. To overcome the continual resistance which it experienced in emptying itself of blood, its action, and consequently its nutrition, increased in proportion. The patches of ossification in the aorta will probably explain, he thinks, the cause of the pulse of one wrist being smaller than that of the other, as it is highly probable, though not specified by the dissectors, that the orifice of one of the subclavian arteries was narrowed, as was the case in a patient whose history is related in one of the former letters, and who presented a similar phenomenon in the pulse.

The paralysis on the left side of the body was preceded by spasmodic contractions in the hand and foot of that side, and the disease of the heart did not prevent the symptoms of cerebral inflammation from following their usual course. Again, the inflammation of the brain did not sensibly affect the symptoms attendant on the cardiac affection.

Our author has remarked in former letters, that in almost all cases of cerebral inflammation, the paralysis confined at first to one side, extended finally to both sides, with loss of sensibility and a state of coma. Dr. Lallemand attributes this series of symptoms to compression of the sound hemisphere from the gradual tumefaction of the inflamed one—an explanation, in our opinion, extremely ingenious and probable.

*Case.* 2. The following case was communicated to our author, while the former was in the press, by Dr. Andral, junior, whose pathological researches are already favourably known to our readers.

A man, aged 27 years, had felt, for some time, a sort of weight rather than pain in the right side of his head, to which he paid little attention. On the 18th December, 1821, after a hard day's labour, he experienced in the left arm a great tremor, soon after which he complained of tinnitus aurium, and then fell into a state of insensibility. 19th. Return of the natural and sensorial functions, but continuation of the spasmodic affection of the right arm. On the 20th, the tremors of the arm changed into a sense of weakness, want of power, and numbness of the same member. 22d. On entering the St. Louis hospital, he presented the following symptoms—viz. pallor of the face—integrity of the intellectual functions—paralysis of the left arm—at least he had little power over this member, although the hand was involuntarily bent on the fore-arm—pain in the right side of the head—pulse slow and feeble—the other functions undisturbed. Some trifling and inactive remedies were employed. The same state continued till the 25th. On the 26th there was redness of the face, strong cephalalgia (12 leeches to that side of the head). 31st. 15 leeches were applied to each side of the head. Delirium and agitation throughout this day. On the 1st of January, the eyes were rolling in their sockets—head agitated by continual movements from one side to the other—left arm completely paralytic—the right arm and leg affected by spasmodic twitchings—pulse (for the first time) frequent—24 leeches to the neck. The patient died next day.

*Dissection*. The tunica arachnoidea on the superior surface of the hemispheres, particularly on the left side, strongly injected. The circumvolutions of the posterior lobe of the right hemisphere flattened, and giving to the finger the sensation of a fluctuation. An incision gave issue to a quantity of pus, and disclosed an irregular cavity, capable of containing the yolk of an egg, separated from the arachnoid membrane by a very thin layer of cerebral substance, and communicating by a kind of fistulous pipe with another cavity, capable of containing a drachm or two of matter. The internal surfaces of these two cavities were lined with a fine and smooth membrane, which could be separated from the surrounding parts, and when macerated in water, shewed a villous and fibrous structure, not unlike the mucous membranes. The surrounding cerebral substance was neither harder nor softer than natural. The thoracic and abdominal viscera were sound.

Our author justly thinks that, from the state of the membrane inclosing the purulent matter, the inflammation must have been of some considerable duration, although there was no other indication of its existence than the sense of weight in the head. In fact, numerous cases attest, that CHRONIC inflammation will destroy a whole hemisphere of the brain, without exciting symptoms of an alarming nature.

The third case is abridged from the 1st volume of our respected contemporary, the Edinburgh Journal, p. 150, and therefore need not be cited here.

*Case* 4. Miss Perrot, 16 years of age, fell, in the latter part of November 1816, from a first floor, and pitched on her head. She lost not her sensibility at the time, but soon after experienced lancinating pains in the whole circumference of the head. Twenty leeches to the temple,—a vein opened in the foot—sinapisms—a large blister to the head. By these energetic measures the pains were dispersed, and, during a month, she did not complain. At that epoch she experienced acute and lancinating pains in the muscles on the back of the neck, and also convulsive twitchings there, which, at first, came on only once a day, but in the course of a fortnight they became almost constant. She was received into the Hotel Dieu on the 12th of January, 1817. The pupils, at this time, were alternately contracted and dilated by the same degree of light—the muscles of the neck were very painful—the pulse was irregular—the face sometimes flushed, sometimes pale—the patient complained much of cold. Sixteen leeches to the neck and behind the ears—bled from the feet—pediluvia—sinapisms. In the day, continually crying, with convulsive movements, and sickness at stomach—could sometimes answer, though with reluctance, distinctly to questions. She died next day.

*Dissection.* On removing the calvaria, both hemispheres of the brain appeared more protuberant than natural, the circumvolutions flattened, indicating effusion in the ventricles. These were filled with serum. The tentorium adhered to the subjacent arachnoid by a very thin layer of coagulable lymph—the tunica arachnoidea, as well as the whole surface of the cerebellum, was exceedingly injected, and in the left lobe was discovered a smooth body, some lines in thickness, and perfectly circumscribed, about the size of a pullet's egg. When completely detached, it was found to be enveloped in several layers of cellular tissue. When opened, it was found to contain a good spoonful of greenish pus, exactly resembling that discharged from an abscess, and without smell. The internal surface of the cyst was smooth, and rather villous. The substance of the cerebellum was somewhat softer than that of the cerebrum—nothing remarkable in the other cavities of the body.

*Remarks.* The symptoms had been so completely dissipated by a rational and active treatment, that no suspicion was entertained of the existence of an abscess. Our author has shewn, by several histories in preceding letters, that it is, when the inflammatory fluxion ceases and the pus is collected into a focus, that the symptoms diminish and the brain resumes its functions, notwithstanding the presence of a foreign body.* Here, as on former occasions, the symptoms of acute hydrocephalus alone characterized the latter period of the complaint; and it was under this denomination that the case was recorded—nor did the dissection belie the prognosis. "The more, says our author, we advance in the study of chronic *alterations of structure* in the brain, the more we shall be convinced that it is rarely by *them* that death is caused, but rather by inflammation or hæmorrhage in the neighbouring parts—most frequently by arachnitis, acute or chronic. In all these cases the character of the original disease disappears, and its existence is seldom suspected."

---

* "Vous voyez que c'est toujours quand la fluxion inflammatoire cesse, quand le pus se réunit en foyer, que les symptomes diminuerent, et que, malgré la presence de ce corps étranger, la substance cerébrale peut reprendre entierement ses fonctions." 38.

*Case* 5. Gabriel Gontaine, 39 years of age, had been subject from his infancy to frequent bleedings from the nose, and discharges of purulent matter from the ears, both of which had been suppressed for two years past, after a violent blow on the head. During the latter period, he had frequently been affected with something like mental aberrations. Three months previous to his entering the hospital, he experienced a violent pain in the *left* side of his head, which had never ceased. During the last five days of this period, he had lost the use of speech entirely, and was quite disordered in his ideas. Presently, paralysis of the *right* side took place, in which state he entered the Hotel Dieu, on the 5th of February, presenting the following symptoms, viz.—hemiplegia of the *right* side—loss of hearing and speech—pulse frequent and feeble—respiration not affected. Some trifling remedies were employed, and this state continued with little alteration till the 17th, when he died.

*Dissection*. The circumvolutions of the hemispheres of the brain appeared flattened, when the calvarium was removed. In the posterior lobe of the *left* hemisphere a large purulent depôt was found, containing nearly four ounces of matter, enclosed in a strong cyst, composed of two membranes that could be easily separated. The cerebral substance in the neighbourhood of the cyst was very soft, and of a yellow colour in some places. The opposite side of the brain was quite sound. No change in the thoracic or abdominal viscera.

*Remarks*. At first sight, our author observes, one would suppose that the inflammation had only commenced about three months previously to the entrance of the patient into the hospital, since it was only during this period that *pain* had been complained of in the left side of the head. But it must be remembered that, after a violent blow, there was a cessation of an accustomed epistaxis, and of a purulent discharge from the ears, followed by some intellectual derangement—and also that the parietes of the cyst were composed of distinct and well-organized membranes. These circumstances will induce us to conclude that chronic inflammation had been going on in the brain for a much longer period than three months, notwithstanding the want of many phenomena which are usually considered to be attendant on phlegmasia of so important an organ as the brain,

---

*Case* 6. This case is quoted from Scultetus, and is that of a soldier, 27 years of age, who received a sabre wound on the back of the head, with lesion of the occipital bone. It was treated as a simple wound, and six months afterwards the man was received into hospital. The surgeon introduced an instrument through the meninges, and even into the substance of the brain, whence issued a quantity of pus. Paralysis of the *right* side came on, on the 20th day after this operation, and the patient died, seven months after the original wound. On dissection, an abscess was found in the *left* hemisphere of the brain enveloped in a thick tunic. It is evident, our author thinks, that the commencement of this organic change was nearly coeval with the sabre wound, although so long unattended by any external symptoms which would lead one to suspect the existence of such growing mischief.

*Case* 7. A young gentleman, a student at Copenhagen, complained frequently of severe tooth-ache, accompanied by a short dry cough, and some redness of the eyes. He had the tooth drawn, but the pain continued, and even extended to the neighbouring parts, especially the os malæ of that side. One day he suddenly fainted, and was a long time in recovering his senses. Next day he was unable to get up, feeling a heaviness of the head, and an insurmountable oppression. The Physicians administered antispasmodics and stimulants; but he had several epileptic attacks, and finally died exhausted, retaining his senses to the last moment. On dissection, nothing unusual could be detected in the thorax or abdomen; but, on examining the brain, an encysted abscess was found deep in the right hemisphere, the size of a pullet's egg, and filled with fetid pus.

Passing over several cases of encysted abscess of the brain, observed or collected by our author, but which are analagous to those we have analyzed, we come to that class which occupies the principal portion and interest of the volume under review.

M. Lallemand observes that caries of the *temporal bone* more frequently produces chronic inflammation of the brain than caries of any other portion of the cranium. Morgagni and Itard, the only authors who have touched with any freedom on this subject, have, in M. Lallemand's opinion, taken up some false notions respecting it. He therefore considers the topic of investigation as not only very important, but almost new, and proposes to trace the connexion of the two diseases from their lightest shades or forms to their most grave and destructive consequences.

We shall find, he remarks, in the records of medicine, and particularly in the periodical journals, examples of acute and chronic otitis, which have terminated suddenly in death, after evincing symptoms of cerebral affection. On dissection, there have sometimes been found collections of pus in the cavity of the tympanum—the dura mater inflamed, thickened, injected, or softened, and detached from the internal surface of the skull,— and yet no alteration in the brain itself capable of explaining the cerebral symptoms. It is possible, that sufficient attention was not, at that period, paid to the colour, consistence, &c. of the substance of the brain, by which we now recognize the existence of previous inflammatory action; for, from the cases which shall be laid before the reader, M. Lallemand hopes to prove, that the brain does participate in these inflammations of the meninges.

*Case* 8. Coindet, in his memoir on hydrocephalus, relates the case of a youth, 17 years of age, who, after suppurative inflammation of the *right* ear, became affected with violent pains in the head, ardent fever, which put on a remitting form, and after four paroxysms terminated in symptoms of acute hydrocephalus, as convulsions, slowness of the pulse, paralysis of the *left* side, dilatation of the pupils, profound coma, and death in the fifth accession or paroxysm.

On dissection, there were found inflammation and considerable softening of the brain opposite to the right temporal bone (petrous portion) and extending to the ventricles, which were filled with serum. This case, he thinks, requires little comment.

He next relates a case by Dr. Abercrombie, in our respected contemporary of the North (for July, 1818) which we need not farther cite here than by remarking that, after a long discharge from both ears, the mastoid cells became carious, followed by pains in the head, gastric affection, and ultimately coma, convulsions and death.

On dissection, the right hemisphere of the brain throughout half its extent was converted into a purulent and pulpy mass, with great effusion into the ventricles.

*Case* 9. Conrard, 20 years of age, after a scuffle, in which he had received several blows, fell ill, and evinced symptoms of low fever, under which he sank on the 20th day. The magistrate, suspecting that death might have been the consequence of the blows which the young man had received, ordered the body to be opened, and the physicians found as follows;— 1st, the brain rather more injected than natural—2nd, between the surface of the brain and the basis cranii a small quantity of purulent effusion— 3d, in the anterior portion of the *right* lobe or hemisphere of the cerebellum a purulent depôt— 4th, the arachnoid opposite to the *right* petrous portion of the temporal bone adherent to the dura mater, and redder than natural. The physicians took some time to consider this case, and in the mean time questioned the parents of the youth, who had the candour to acknowledge that their son had suffered a long time from pains in the head, and also in the *right* ear. The physicians then renewed their investigations, and found the mastoid cells of the right side full of pus, as well as part of the internal ear. They therefore declared that disorder existed anterior to the scuffle, which could only have hastened the march of the disease.

M. Itard, in his monograph on diseases of the ear, appears to regard a discharge from the meatus externus as a constant symptom attendant on chronic otitis—at least, he recites no case in which it was absent. But M. Lallemand has seen two instances in which there was no discharge. Both of these people had a dull pain in the interior of the ear, aggravated by cold or moisture—an habitual bad taste, and sense of disagreeable odour in the back part of the mouth, especially when the pain was considerable, and in certain positions of the head, or acts of deglutition,—occasional dislikes to food—nausea, and even vomitings.

*Case* 10. Elizabeth Erot, 23 years of age, had had a discharge from the left ear, from the age of 7, (at which period she suffered from the small pox) accompanied by pains in the head, which increased rather than diminished with her age. In the month of November (being then in a late stage of pregnancy) she experienced such severe pains in the crown of her head, as to make her cry out, and which pains were diminished by pressure. At this period the discharge from the ear had diminished. The bowels being constipated, lavements were prescribed, and fomentations to the ear. The pains increased, with spasmodic twitchings of the arms. Her accouchement now took place, but produced no relief, and this interesting female sunk under her afflictions.

On removing the calvarium, the dura and pia mater were found inflamed, and in the left hemisphere of the brain an encysted abscess. The petrous portion of the temporal bone was carious and black. This case is recorded by Bonetus. Our author remarks that the lymphatic temperament is peculiarly prone to chronic affections of the ear, and that the small pox is a very frequent exciting cause of deafness, and purulent otorrhœa. It may be observed, that as the discharge diminished, in proportion was the head-ache augmented. Was this merely accidental? Our author is far from thinking so. He is disposed to view the affair thus :—the chronic otitis produced, by its immediate vicinity, an inflammation of the brain; hence resulted an encysted abscess—and when the envelopes of the brain took on *acute* inflammation, cephalalgia, convulsions, suppression of the discharge, ensued.

Our author next cites two cases from the writings of Dr. Abercrombie, with which our readers are sufficiently acquainted.

*Case* 11. (From Sabatier). A small ball of paper introduced into the ear was seen by Mr. Sabatier, to produce disastrous consequences. The efforts to extract the foreign body only served to force it deeper into the ear—nevertheless the patient continued to enjoy good health for some months, at the end of which time he was seized with what was termed putrid or malignant fever, accompanied by violent pains in the head, of which he died on the 18th day. On opening the head it was found that there was an adhesion of the brain to the dura mater covering the petrous portion of the left temporal bone, and at this place was situated an abscess, communicating with the cavity of the tympanum, where the foreign body was lodged.

*Case* 12. (From Labius.) Jean André, robust, and in the prime of life, experienced about the end of April, 1813, an attack of continued fever, accompanied by grave symptoms, from which he recovered, but still remained melancholy, with defective vision, which last symptom increased till he completely lost his sight, having several times, however, partially recovered vision, during temporary discharges of purulent matter from the ear. Towards the end of the summer of the said year, a tumour arose behind the left ear, which lessened a little during a discharge from the meatus, and enlarged again a few days afterwards. These alternations took place three or four times, accompanied by periodical pains in the head. Finally, on the 1st of November of that year, the patient experienced an attack of apoplexy, in which state he lay insensible and motionless during three days. On the fourth day, he recovered the use of speech, and asked for something to eat. He swallowed with great difficulty. The *left* side was paralysed—the *right* agitated by convulsions to the moment of his death, which took place on the 11th of November.

*Dissection.* The vessels of the dura mater were distended with blood, and from the brain, opposite to the *left* ear, there issued nearly five ounces of pus. All the rest of the brain was sound, the vessels being much dilated. The petrous portion of the temporal bone was carious, and a communication existed with the external ear.

The question here occurs, did the inflammation, in this case, commence externally or internally? M. Lallemand thinks it improbable that an inflammation of the brain should extend itself to the temporal bone, in such a manner as to form for itself an opening into the ear—whereas we have numerous instances on record, to shew how readily an inflammation of the ear produces caries of the bone, and spreads to the membranes and substance of the brain.

Our author next quotes a case from the Medico-chirurgical Journal (1st series, Oct. 1819) as extracted from the Dublin Hospital Reports, and there published by Dr. O'Brien of Dublin. We need only refer our readers to the original case, or to our analysis of it in the number above-mentioned.

*Case* 13. This case is quoted from Sedillot's Journal, vol. 45, and has been communicated (to some of the English periodicals, we suppose) by our distinguished countryman, Mr. Brodie.

A youth, about fifteen years of age, subject to vertigo from his infancy, learnt with difficulty, but retained what he learnt with wonderful tenacity, and appeared endowed with a sound understanding. At the age of two years, he became deaf of the left ear, in which a suppuration and discharge were established. In 1809, he being then in his 15th year, there appeared a small fungous excrescence at the bottom of the meatus externus, to which the unguentum nitratum was applied, with the effect of stopping the discharge in 15 days. At this period commenced an acute pain in the head and ear. The ointment was discontinued—the discharge returned, and the pains ceased. Some time afterwards, the ointment was reapplied, and the discharge again interrupted. On the 8th day after the cessation of the discharge, the boy became affected with such acute pain in the head, that he was forced to cry aloud, and said he would certainly go mad. In the course of a few days he suddenly became insensible, with dilatation of the pupils, slowness of the pulse, and other symptoms of cerebral compression. He died in a state of coma.

*Dissection.* Vessels of the dura mater gorged with blood, as also those of the pia mater and arachnoid, which was quite dry on its surface. Two ounces of serum in the ventricles. In the *left* hemisphere of the brain, a cyst was found three inches in diameter, consistent and vascular, containing thick purulent matter. The inferior extremity of the cyst rested on the petrous portion of the temporal bone, and a small opening of communication between the ear and the cyst existed, the bone being carious. The cerebral substance surrounding the cyst was yellow, and much softer than natural.

*Case* 14. Our author next quotes a case, as published by Dr. Parkinson in the MEDICAL REPOSITORY for March 1817, under the title of *Hydrocephalus*, but which M. Lallemand very properly thinks might have received a more appropriate designation. The patient was a youth of 14 years, who had, for some time, experienced violent accessions of pain in his head and right ear, from whence there was sometimes a discharge of purulent matter. This discharge increased, and became both fetid and sanguinolent. The little patient got weaker and weaker, had attacks of convulsions, fell into a state of insensibility, with dilated pupils, slow pulse, &c. in which state he died. On dissection, three ounces of fluid were found in the ventricles, and a small abscess in the middle lobe of the right hemisphere of the brain, communicating with the ear by a carious aperture in the temporal bone, through which a sound could be introduced into the meatus externus.

*Case* 15. (From M. Itard). G. R. aged 22 years, being harrassed by a severe tooth-ache, had several attempts made by a surgeon to extract the tooth, but without success. These attempts exasperated the tooth-ache—fever supervened—and a more expert surgeon being found, the tooth was removed. But, the febrile symptoms and pain still continuing, the patient was sent to the hospital, on the 6th of October. The pulse was now full and bounding—there was some delirium. Bleeding and some other means were employed. On the 10th October, a purulent discharge from the ear took place, but the symptoms continued, and the patient died on the 4th of November.

*Dissection.* Besides several marks of inflammatory action in the brain, the auditory nerve of the right side, both portio mollis and portio dura, was found in a state of suppuration, and a quantity of pus collected in the meatus auditorius internus and externus.

*Case* 16. (From the same). Peter Remy, aged 60 years, an hæmorrhoidinarian for 40 years, was taken in the spring with a slight sore throat, which was soon dissipated; but, in a few days, succeeded by a most severe pain in the right ear, unmitigated by fomentations and other anodyne applications. After three days of inexpressible sufferings, a purulent discharge took place from the ear, which brought relief to the pain, and this discharge continued to flow, for twenty-one months, with some temporary interruptions, during which the pain returned. After this period, the patient began to experience some formidable symptoms, as sense of weight in the head, nausea, furred tongue, fetid breath, small pulse, deafness on the right side, from the ear of which side, a very acrid, fetid, and corrosive discharge issued. This discharge abated, or even stopped occasionally, and then the head-ache increased, or delirium occurred. At length the patient sunk, with all the symptoms of cerebral affection.

On dissection, there was found a considerable quantity of pus in the affected ear—the sinuses of the dura mater were gorged with blood—the latter member, where it covered the petrous portion of the temporal bone, was thickened, discoloured, and pierced in several places. There was much fetid pus between the dura mater and bone, and an encysted abscess, containing inodorous pus, in the middle lobe of that hemisphere. The bone itself was carious, and all the internal parts of the ear disorganized, and full of pus.

M. Lallemand thinks that all the symptoms of this case go to shew, that inflammation commenced in the ear, and spread from thence to the envelopes and substance of the brain.

*Case.* 17. M. Le Blanc, being at the mint, had the imprudent curiosity to place his head so close over one of the moulds into which a man was pouring melted silver, that he felt like a sudden electric shock, that deprived him of sensibility, and was followed by constant head-ache, and sense of weight about the brain. On the 6th day there was complete deafness. On the 8th, violent cephalalgia, and sensation as if the bones of the skull were forcibly separating, with fever, hard pulse, &c. During the next seven days, the patient was bled eleven times from the arm and jugular vein, with temporary relief. Till the 15th day, the pains were so violent, especially in the evenings, as to occasion spasmodic movements in the muscles of the face, and in the arms and lower extremities—insomnium—subsultus tendinum—small pulse—sense of extreme weight in the head. In the course of the next fortnight, there was a slight diminution of the pain in the head, the other symptoms continuing the same. The seat of this pain appeared to Le Blanc himself to indicate the existence of a purulent depôt between the dura mater and the left parietal bone, and he became convinced that there was no other chance of delivery than by trephining the part. Lecat, his friend, was summoned for this purpose; but an hour before his arrival, the patient fell (for the first time during nearly 56 days) into a sound sleep, on awaking from which, he found his pillow covered with pus, which had drained from the left ear in a stream, and gave complete relief to the patient's sufferings. During fifteen days the discharge was constant and considerable; but after this period it gradually decreased, although it required more than two years of convalescence to restore him to health. During this convalescence, there was more or less of discharge from the ear, and whenever, by accidental circumstances, this discharge was arrested, there arose a train of unpleasant symptoms that threatened serious consequences.

*Case* 18. Joseph Prevot, 40 years of age, received a violent blow on the right cheek, for which he was treated at the St. Louis hospital, and soon discharged as cured. Nine months afterwards, and without any apparent cause, he began to experience acute pain in the forehead, followed by delirium. He was bled, and had an emetic administered, and in about a fortnight, 17th July, he was sent to the Hotel Dieu. The delirium was violent, agitation extreme, face flushed, conjunctivæ inflamed, tongue dry and rough, skin hot, pulse hard. Bled to 12 ounces. 18th. The symptoms increased in degree. Leeches to the temples, and another general bleeding. 19th. Coma, which continued till the 23d, when the patient died.

On dissection, the arachnoid membrane was found adhering by a gelatinous layer to the dura mater—the lateral ventricles dilated to double their natural size, but not containing much water—the lining of the ventricles covered with granulations—at the inferior and posterior part of the cerebellum, between the pia mater and arachnoid, there was a tumour the size of a small egg, enclosed in a cyst, and containing several grumous clots of blood in a decomposed state. The surrounding portion of brain was of a greenish yellow colour, and the adjacent bone was carious.

*Case* 19. (From professor Frank.) Jean Otto, 26 years of age, experienced for several years, an obstruction or embarrassment in the head, and particularly in the nose, accompanied with acute pain extending towards the right temple. After some time, a discharge began to take place from the nostril of that side, consisting of sanious matter mixed with blood. In about nine months afterwards, a thin lamella of bone was discharged, but pain in the head, and swelling of the nose continued, and a discharge began to issue from the other nostril, from which also a carious piece of bone was thrown off. Paralysis of the left side next took place, and the patient died in a few days.

The upper part of the brain was in a good state, but in the lateral ventricles was found a thick ichorous matter. Before the sella turcica and under the decussation of the optic nerves, there was a perforation of the skull that would admit a nut, and filled with purulent matter, which had communication also with the nose.*

We have now presented our readers with the principal facts or cases contained in this volume, and have only to take some notice of the observations which M. Lallemand has added in conclusion.

He thinks it results from the cases narrated, in this and the preceding letters, that in abscesses of the brain (provided the patient survives long enough) a cyst is organized around the pus, as around any other foreign body which is placed long in contact with living tissues, as, for example, in cerebral hæmorrhages, gunshot-wounds, &c. It is reasonable to suppose that the period at which a cyst begins to become organized, in these cases, varies according to the more or less rapid march of the inflammation; but it is always to be borne in mind, that chronic inflammations often produce extensive alterations in the cerebral mass, without exhibiting any symptoms that might lead to a suspicion of their existence. Nevertheless, where the cases have been carefully observed, and the symptoms and dissections accurately noted, our author has found a considerable relation between the duration of the disease and the progress of the organization in the cyst. For instance, in a case where death took place thirteen days after the first symptoms appeared, the parietes of the purulent depôt were already lined with a soft and somewhat vascular membrane. In another case, of eighteen days, the same thing was observable. At the end of 37 days, the cyst was white and easily lacerable, resembling concreted pus. At 53 days, the membrane was soft and vascular, more distinct than in the other cases, but not sufficiently tenacious to be dissected out without laceration. At the end of two months, the cyst was smooth, perfectly circumscribed, presenting exteriorly several layers of cellular substance, and internally exhibiting a soft mucous surface, resembling that of an old abscess in any other part of the body. At three months, the sac, still more vascular, had a denser structure, and a more considerable thickness. Finally, after some years duration, the exterior presented several cellular layers—the centre a

* A nearly similar case was opened some time since, by Mr. Baxter and the Editor of this Journal. Mr. B. it is hoped, will publish the case.

dense tissue, thick, and apparently fibrous—internally, all the features of a mucous membrane, somewhat inflamed.

It appears then, that the work of organizing a barrier around a purulent depôt, may be carried on for years with uninterrupted activity. Is it then, says our author, to be wondered at, if this foreign body should become a constant source of irritation and of determination of blood to the parts, inducing ultimately an organic alteration in the neighbouring structures.

1. M. Lallemand observes, that hitherto little importance has been attached to the alterations produced in the neighbourhood of the encysted abscess; and yet, he thinks, that death is rarely occasioned by the said abscess alone; as it is, in fatal cases, almost always accompanied by acute inflammation of the surrounding cerebral substance, a chronic inflammatiou of the arachnoid covering,

2. Here arises a question—Is a cure possible, in the event of an encysted abscess of the brain?—Or, in other words, is the pus contained in such an abscess ever absorbed? This is a problem which, perhaps, can never be solved by direct observation. Analogy, in cases of cerebral hæmorrhage, would lead us to expect, that absorption is not impossible; but the structure of the lining membrane of the purulent collection (being that of a mucous membrane) must render it more disposed to exhalation than absorption in general—more likely to augment than diminish the contents of the cyst. Neither are these kinds of structures capable of forming adhesions one with another, even if their contents were absorbed.

3. To what our author has said in former letters, respecting the general causes of chronic inflammation of the brain, he has nothing to add in this, excepting caries of the skull, and especially caries of the temporal bone. This cause, he considers to be so common, so powerful, and so little suspected, that he has thought it right to dedicate a large portion of the letter before us, to its investigation, and to diseases of the ear in general.

Acute otitis, he remarks, is rather more frequently seen before than after puberty; but both sexes and all temperaments seem equally exposed to its causes. External otitis is often produced by the extension of some cutaneous affection to the mucous membrane of the meatus externus—more particularly in variola. In such cases, the inflammation very readily spreads to the interior of the ear, too often inducing caries of the bones, permanent deafness, or fatal affections of the brain itself. Among the accidental causes, cold, applied suddenly to the ear, through the medium of a stream of air, is one of the most common. Internal otitis very frequently takes place in the latter stage of fevers—not, he observes, as a critical turn of the disease, but because fever is, in fact, inflammation of the brain, and the ear, being so contiguous an organ, is more frequently found to suffer than any other.

External otitis, less formidable than internal, is distinguished by the suddenness of the discharge after the pain has commenced. On the second or third day, the lining membrane of the meatus externus is red, tumefied, and covered with pus, or a puriform secretion. In internal otitis, on the other hand, the lining of the meatus continues dry during several days, and at length the discharge comes on all at once, and is very profuse. This discharge, in such cases, makes its way through the eustachian tube, and continues to flow through that channel.

Internal otitis, is often accompanied by symptoms similar to those appertaining to inflammation of the brain or arachnoid membrane— with which, indeed, it is not seldom complicated, so as to render the diagnosis extremely difficult. Thus, in otitis, the pain is not always confined to the ear, but sometimes extends to the whole head, being more or less violent, lancinating, and compressive. The connexion of the portio dura of the auditory nerve with so many other nerves, may explain the spasmodic affections, and many other symptoms, accompanying inflammation of the internal ear.

Otorrhœa, or chronic catarrh of the ear, is often the consequence of otitis acuta. But in whatever way it commences, it generally ends by affecting equally the meatus externus, and the membrana tympani. The mucous membrane becomes thickened, red, and sometimes bloody—the cavity of the meatus diminished—the membrana tympani destroyed or perforated, as shewn by the passage of air or other fluids through the ear. The disease too often ends in caries of the bone, and inflammation of the brain or its membranes. The physician, therefore, who is called in to treat this disease, should employ the most energetic means from the very beginning, if he wishes to ward off the fatal catastrophe which follows, if these means be not used. To otorrhœa our author has found the scrofulous constitution most prone, as also those constitutions which have been much affected with cutaneous diseases. The smell, colour, and consistence of the discharge vary much in different individuals, and in the same individual under different circumstances. It is generally diminished under the influence of a dry and warm temperature, exercise, and low living. In simple cases, it will entirely disappear under these. It is easily renewed or augmented by the reverse of the above, and especially by cold and moisture, too much intellectual exertion, and excesses of the table.

Sometimes the sudden suppression of the discharge is purely mechanical, as, for instance, when crusts form at the bottom of the meatus, from desiccation of the viscous matter—when ointments are crammed in, and form plugs, confining the discharge—or, finally, where polypous vegetations present an obstacle to the egress of the purulent secretion. In all these cases, if the matter does not make its escape by the eustachian tube, there arises a sense of tension, weight, and pain—and sometimes symptoms of compression of the brain.

Occasionally, the discharge from the ear ceases, in consequence of some other operation going on in the system, as the epoch of puberty, pregnancy, &c. at others in consequence of some pathological fluxion or determination to a particular organ. Thus, our author has seen these discharges alternate, with attacks of rheumatism, catarrhus vesicæ, leuchorrhœa, &c. In some cases, the new disease is so violent, that it may be desirable to re-establish the aural discharge; but this, he thinks, can seldom be necessary, or safe. It is much better, he observes, to institute another drain by seton, and to adopt a rigid antiphlogistic system of treatment. The most dangerous metastasis, is that to the membranes or substance of the brain. When this has lasted but a short time, terminating quickly in death, no trace, he avers, of its existence, may be cognizable on dissection. When the inflammation has lasted but a few days, a softness only will be found in the brain, generally corresponding with the petrous portion of the temporal bone. When prolonged beyond this period, an abscess will be usually detected, the matter of which is fluid in the centre, and pulpy at the circumference. But it is not always in the corresponding hemisphere that this abscess exists—sometimes it is in that opposite to the diseased ear—proving, he thinks, a real metastasis, and not merely an extension of the disease of the brain. Inflammation of the arachnoid, he has rarely found to terminate by adhesion, but by a serous or sanguineous effusion, with some alteration in the texture of the membrane itself.

Otorrhœa then, however benign at the beginning, if neglected or maltreated, generally goes on from a mucous to a purulent, and ultimately a thin sanious discharge, of that peculiar fetor which always accompanies caries of a bone. The fragments of ossicula auditus come away first, and then small particles of the temporal bone itself. The affair is now very serious. There is another kind of otorrhœa, observes our author, more rare more insidious, and almost unknown—it is that which takes place through the eustachian tube. The patient experiences a dull pain in the region of the ear, sometimes fixed, at other times shifting about—sometimes constant, at others intermitting. He feels a tinnitus aurium—hears a continual buzzing noise, like that of a mill, or a water-fall—is hard of hearing, and afterwards becomes quite deaf for a time—then recovers the auditory powers, with the noises above-mentioned. This loss and recovery of the auditory functions, depend, he thinks, on the accumulation in, and discharge of matter from the tympanum. The patient has a bitter taste in his mouth, a fetid breath, occasional nausea, or vomitings of fetid matter—with expectoration of the same in violent fits of coughing. He also takes a distaste against, or even nauseates his food—loses his appetite—becomes despondent, and emaciates from day to day, without his medical attendant knowing why. Generally, these symptoms are attributed to an affection of the stomach or lungs. Medicines are given with this view, but, of course, without effect. The caries goes on—the membranes or the brain become affected, and death closes the scene.

Sometimes the sanious otorrhœa is accompanied by firm, yet fungous excrescences, which frequently bleed on the least touch. The attempts to cure these by extraction, cauterization, caustics, or desiccative ointments, generally increase the evil, and produce disastrous consequences. These vegetations are prolongations of the membrane of the tympanum, or of the dura mater itself. They are produced by the same causes which produce caries, and resemble those fungosities which are developed on the surfaces of carious bones. They are to be distinguished usually from polypi, by the symptoms which have preceded them, and by the nature of the discharge attending them. Polypi are commonly accompanied by an abundant discharge; but it is of a mucous nature, and does not discolour the silver probes, or other instruments.

In pursuing the ravages produced by caries, it will be seen that it (caries) does not affect indiscriminately all parts of the temporal bone—but, on the contrary, that it follows the direction of the different conduits that have relation with the cavity of the tympanum. Indeed, when we reflect on the numerous cavities and canals in the temporal bone, lined with a continuation of the same membrane, we cannot wonder at the frequency of caries, even in its petrous portion. Of all parts, however, the mastoid process is the most frequent site of caries, and after that the petrous portion of the bone, in the vicinity of the semicircular canals, and vestibulum.

When the caries has once penetrated to the dura mater, or the inflammation of this membrane has extended to the arachnoid, or brain, then a fluxion or determination is made towards the centre of the sensorium, which, not uncommonly, suspends that which has previously existed towards the ear. Then it is that the discharge ceases, or becomes notably diminished. Dissection shews the dura mater destroyed, covered with pus, or lying in contact with an abscess.

We have now turned over the last page of the work before us, and have endeavoured to compress into a moderate article, almost the whole substance of the book. M. Lallemand does not enter upon the treatment of this insidious and dangerous disease, but refers to M. Itard's work, as the best on the subject of aural diseases. We have seen several cases that might have been reported here with advantage, but we hope the gentlemen themselves who had the immediate charge of them, will lay them before the public. We think we have said enough to put the reader on his guard, when he has a disease of this description to deal with, and that this short article will stimulate him to a more careful observation of otitis and otorrhœa than he has formerly been in the habit of bestowing.

